# Genomic epidemiology and characterization of *Staphylococcus aureus* isolates from raw milk in Jilin, China: evidence of contamination with *optrA*-positive, *cfrA*-positive, and *poxtA*-positive strains and human–livestock transmission

**DOI:** 10.3389/fmicb.2025.1547283

**Published:** 2025-05-29

**Authors:** Qianhong Liu, Pengming Ma, Qiaoyi Gu, Wen Yang, Chunhua Li

**Affiliations:** ^1^College of Animal Science, Xichang University, Xichang, China; ^2^Key Laboratory of Animal Epidemic Disease Detection and Prevention in Panxi District, Sichuan, China; ^3^Xichang Agriculture and Rural Bureau, Xichang, China; ^4^College of Animal Science and Technology, Jilin Agricultural Science and Technology University, Jilin, China

**Keywords:** raw milk, *Staphylococcus aureus*, MRSA, drug resistance, genetic characteristics, whole genome sequencing

## Abstract

Jilin province is the primary region for dairy cow and milk production in China. However, there are few reports on the genetic characteristics, antibiotic resistance, and prevalence of *Staphylococcus aureus* (*S. aureus*) in raw milk. Between 2021 and 2022, researchers identified 214 *S. aureus* strains, with a prevalence rate of 42.8% in cattle farms across Jilin province. Among the 214 strains, 22 isolates of methicillin-resistant *Staphylococcus aureus* (MRSA) were identified, with 13 exhibiting prevalent antibiotic resistance. Penicillin exhibited the highest resistance rate (145/214, 67.76%). The profiles of drug resistance, pathogenicity, genetic traits, and biofilm formation were examined through whole genome sequencing (WGS) of 44 isolates, comprising 22 MRSA strains and 22 methicillin-sensitive *Staphylococcus aureus* (MSSA) strains. Of the 44 isolates, 25 (56.82%) exhibited multiple resistance, while 31 (70.45%) demonstrated drug resistance characteristics. We identified 12 distinct types of drug resistance genes, including those associated with tetracycline, quaternary ammonium salt disinfection, and *β*-lactam. Six strains were found to carry genes conferring resistance to linezolid. The *Aur* and *hlgA/B/C* virulence genes were identified in at least 90% of the strains, with ST9-t899 emerging as the predominant type. Human–livestock transmission may be present, as indicated by the single-nucleotide polymorphism (SNP) and WGS data. The isolates also exhibited increased resistance. Moreover, strong biofilm formation was observed among the MRSA strains compared to the MSSA strains. The isolates exhibited multi-drug resistance, a broad spectrum of drug resistance, and various drug resistance phenotypes. CC398 and CC9 demonstrated potential for human-to-livestock transmission, as evidenced by the identification of 22 CC9 and 15 CC15 strains among the 44 isolates. *OptrA, cfrA,* and *poxtA* genes were identified in five, seven, and one strains, respectively, indicating contamination within the population. Given that raw milk is a fundamental food source, it is essential to monitor the molecular characteristics and antimicrobial resistance (AMR) of *S. aureus* to ensure food safety and hygiene. From a One Health perspective, controlling antimicrobial resistance (AMR) is crucial as it can be transmitted from food-producing animals to humans, thus impacting public health.

## 1 Introduction

*Staphylococcus aureus* (*S. aureus*) is associated with various infections in animals and livestock. It has the potential to contaminate both food and the environment (Suzuki and Tagami, 2015; Liu et al., 2024; Kurz et al., 2024). As an opportunistic pathogen, it can infect humans through direct interaction with animals, consumption of contaminated food, or exposure to the environment. It can cause skin and soft tissue infections, bacteremia, endocarditis, and potentially death (Kadariya et al., 2014; Asati et al., 2024; Ahmad-Mansour et al., 2021). Methicillin-resistant *Staphylococcus aureus* (MRSA) is a prominent multidrug-resistant pathogen commonly found in healthcare settings, capable of colonizing or infecting both humans and animals.

Livestock-associated MRSA (LA-MRSA) has become a growing concern. Lineages such as ST398 and ST9 are linked to zoonotic transmission, contributing to the growing burden of antimicrobial resistance (AMR) in humans (Aires-de-Sousa, 2017; Cuny et al., 2015; Matuszewska et al., 2020). LA-MRSA plays a significant role in the increasing prevalence of MRSA, resulting in an increase in hospital-acquired infections. It can also colonize animals and individuals in close proximity to one another. Transmission occurs through animal-derived food, contaminated environments, and specific occupational settings (Aires-de-Sousa, 2017; Cuny et al., 2015; Matuszewska et al., 2020). Numerous studies have examined MRSA in porcine populations. Nonetheless, there is limited knowledge regarding its prevalence, drug resistance, and genetic characteristics in milk. The hygiene standards of individuals handling milk, the surrounding environment, and the animals involved are contributing factors. These conditions increase the likelihood of human infection with drug-resistant bacteria, posing a significant threat to human health. *S. aureus* synthesizes toxins, including enterotoxins, that remain functional even at elevated temperatures. This creates significant food safety challenges (Ferri et al., 2017; Ts et al., 2023; Loffler and Tuchscherr, 2021; Sarwar et al., 2023).

The government of Jilin province is actively promoting the expansion of the dairy industry. However, there is limited information regarding the emergence and molecular characteristics of *S. aureus* isolates in cattle. From 2021 to 2022, we collected 500 raw milk samples from intensive and household cattle farms in Jilin. The analysis focused on drug resistance, genetic traits, molecular features, and biofilm formation in *S. aureus* isolates. This study offers guidance on the prudent use of antibiotics and contributes to ensuring food safety. The emergence of antimicrobial resistance in animal-derived foods represents a quintessential “human–animal–environment” challenge within the One Health approach. Therefore, monitoring raw milk is crucial.

## 2 Materials and methods

### 2.1 Sample collection

We collected 500 samples from two large cattle farms, each containing between 800 and 5,000 cows. Samples were also collected from two small household farms housing between 100 and 500 cows. Large farms utilize machinery to milk cows three times daily, while small farms rely on manual milking, conducted three times daily. Data were collected by researchers in the Jilin region between 2021 and 2022. Convenience and random sampling criteria were employed for data collection. Samples were obtained from fresh milk, the surrounding environment, and personnel at the cattle farms. The participant group included managers, breeders, veterinarians, dairy workers, gatekeepers, and interns (see Table 1). A 50-ml sterile centrifuge tube was used for obtaining the milk sample. The specimen was maintained at a low temperature and transported to the laboratory within 12 h. Surface samples were obtained from various objects. Initially, we disinfected railings and other surfaces using sterile cotton swabs. Subsequently, the swabs were placed in a sterile tube containing 1 mL of 7.5% sodium chloride broth culture medium. Air sample collection involved opening the lid of the medium and exposing it to air for approximately 1 h. The sample was incubated at 37°C in the laboratory to monitor bacterial growth. A sterile nasal swab was meticulously inserted into the personnel’s nostril until resistance was felt. The swab was rotated in three full circles, temporarily held in place, and then immersed in the sterile tube containing 1 mL of 7.5% sodium chloride broth culture medium.

**Table 1:** Number of samples.

Farm	Source		Number	Code
Intensive cattle farm 1	Raw milk		170	CF, CP, CF, EF, FF
	Staff		6	DP
	Environment	Railings in adult cattle area (health zone)	2	
		Railings in adult cattle area (postpartum period)	2	
		Water tank in dry cow area	2	
		Railings in nurturing cattle areas (6–12 months)	2	
		Railings in nurturing cattle areas (13–15 months)	2	
		Railings in high-yielding cattle area	2	
		Sweeping cart railing	2	
		Milking hall railing	2	
		Vegetable soil	2	
		Milking parlor air	2	
Intensive cattle farm 2	Raw milk		170	SA
	Staff		6	
	Environment	Railings in adult cattle area (health zone)	2	
		Railings in adult cattle area (postpartum period)	2	
		Water tank in dry cow area	2	
		Railings in nurturing cattle areas (6–12 months)	2	
		Railings in nurturing cattle areas (13–15 months)	2	
		Railings in high-yielding cattle area	2	
		Sweeping cart railing	2	
		Milking hall railing	2	
		Vegetable soil	2	
		Milking parlor air	2	
Household cattle farm 1	Raw milk		50	L
		Living quarters	4	
Household cattle farm 2	Raw milk		50	G
		Living quarters	4	

### 2.2 Sample isolation and identification

The newly collected samples were incubated in 7.5% NaCl broth (Qingdao Haibo Biotechnology Corporation, China) for 24 h at 37°C. The samples were subsequently cultured on mannitol salt agar (Qingdao Haibo Biotechnology Corporation, China). The yellow colonies were subsequently cultured on Baird-Parker agar for 16 h at 37°C (Qingdao Haibo Biotechnology Corporation, China).

Clones were identified using PCR targeting the 16S *rRNA*, *nuc,* and *mecA/mecC* genes, and positive isolates were subsequently stored at −20°C. Supplementary Table S1 presents the primers utilized in this study. The PCR protocol was conducted according to the method described in the literature (Suzuki and Tagami, 2015). The PCR amplification system is presented in Supplementary Table S2. The identification of isolates as *S. aureus* was confirmed only when both 16S *rRNA* and *nuc* genes tested positive.

### 2.3 Antimicrobial susceptibility testing

The Clinical and Laboratory Standards Institute (CLSI) established criteria for evaluating 13 antimicrobial agents against *S. aureus* using disc diffusion methodology. The tested agents included penicillin, ampicillin, oxacillin, chloramphenicol, gentamicin, tetracycline, erythromycin, rifampicin, vancomycin, clindamycin, ciprofloxacin, levofloxacin, and linezolid. ATCC25923 served as the positive control in our laboratory.

### 2.4 Whole-genome sequencing (WGS)

#### 2.4.1 Extraction DNA

The isolates were cultured in brain heart infusion (BHI) broth (HopeBio, Qingdao, China) at 37°C. Genomic DNA was extracted and purified from all isolates utilizing a bacterial DNA extraction kit from Sangon Biotech, China. The quantity and quality of the DNA were assessed through agarose gel electrophoresis and fluorometric analysis. Subsequently, the samples were sequenced by Novogene Company in Beijing.

#### 2.4.2 Whole-genome sequencing

The sequencing libraries were generated using the Illumina platform, which is recognized for its reliability. Invalid data were filtered out. The original image data from sequencing were converted into sequence data through base calling. The raw data were generated with a coverage of at least 100-fold. We excluded low-quality bases, any undetected bases (designated as N), and joint information from the raw data. The resulting data were referred to as clean data. After examining the sequencing data, base mass distribution, and base content distribution, we utilized the cleaned data for further analysis.

#### 2.4.3 Assembly and annotation of the genomes

The raw data were processed to derive clean data. Genome assembly was facilitated using SOAP denovo (version 2.04), SPAdes, and ABySS. Various K-mers were utilized, each with distinct default values for the assembly process. The assembly results from the three software programs were integrated using CISA software. The assembly outcome that satisfied the minimum criterion was selected. The gap-closing software Pilon was used to address the deficiencies in the assembly outcomes. Sequences with low depth, defined as less than 0.35 of the average, were eliminated, and fragments shorter than 500 bp were discarded. In addition, contamination was removed to achieve the final assembly outcome. Gene prediction and annotation were performed using the RAST server (Liu et al., 2024).

### 2.5 Molecular characterization and phylogenetic analysis

The WGS data were utilized for multiple analyses. This encompassed MLST typing, spa typing, and the assessment of antibiotic resistance genes (ARGs) and virulence factors. These resources were accessed through the Centre for Genomic Epidemiology website.^1^

The phylogenetic tree was constructed using single-nucleotide polymorphisms (SNPs) and WGS through CSIPhylogeny. MRSA strains of CUHK_BJ2002 were used as reference strains.

### 2.6 Biofilm formation assay

The biofilm quantification assay has been extensively documented in the literature (Kurz et al., 2024; Kadariya et al., 2014). A total of 44 isolates were collected from TSB overnight. The cultures were subsequently diluted with TSB to achieve a density of 0.04 at 600 nm (OD_600_). Following a 4-h cultivation at 37°C with 200 rpm, the cultures were subsequently transferred to 96-well plates for a 24-h incubation at 37°C. The wells were washed three times with phosphate-buffered saline (PBS) and subsequently fixed with methanol for 15 min. Following the removal of methanol, the bacteria were subjected to staining with 0.2% crystal violet for a duration of 30 min, after which they were washed three times with PBS. The bacteria were exposed to 33% acetic acid for a duration of 10 min. Subsequently, the value was measured using an ELISA reader at a wavelength of 595 nm. *S. aureus* NCTC8325 served as the positive control, while TSB functioned as the negative control. The cut-off value was established at 0.4, defined as Dc = 2 times the average of the negative control. An OD_595_ value exceeding 1.73 indicated robust biofilm formation. When OD_595_ ranged from 1.73 to 0.4, biofilm formation was classified as moderate. An OD_595_ value below 0.4 indicated weak biofilm formation. The assay was conducted three times.

### 2.7 Transferability experiment of *poxtA, optrA,* and *cfr*

Plasmid DNA was extracted from multiple strains: MRSA containing the *poxtA* gene (FF45), methicillin-sensitive *Staphylococcus aureus* (MSSA) with the *optrA* gene (SA27), and MSSA harboring the *cfr* gene (SA12). This adhered to the guidelines provided by the Midi kit from Sangon Biotech, China. The purified plasmids were then transformed into the *S. aureus* strain RN4220 via electrotransformation using the Gene Pulser Xcell from Bio-Rad. The isolates contained *optrA* and/or *cfr* (Asati et al., 2024). The transformants were incubated on brain heart infusion agar (Qingdao Haibo Biotechnology Corporation, China) supplemented with 10 mg/L florfenicol for 24 h. Following the identification of the *poxtA* gene in the transformants via PCR, the antimicrobial susceptibility of these transformants was assessed using the previously established method. The primers utilized are listed in Supplementary Table S1.

## 3 Results

### 3.1 Resistance profile of *Staphylococcus aureus*

**Figure 1:**
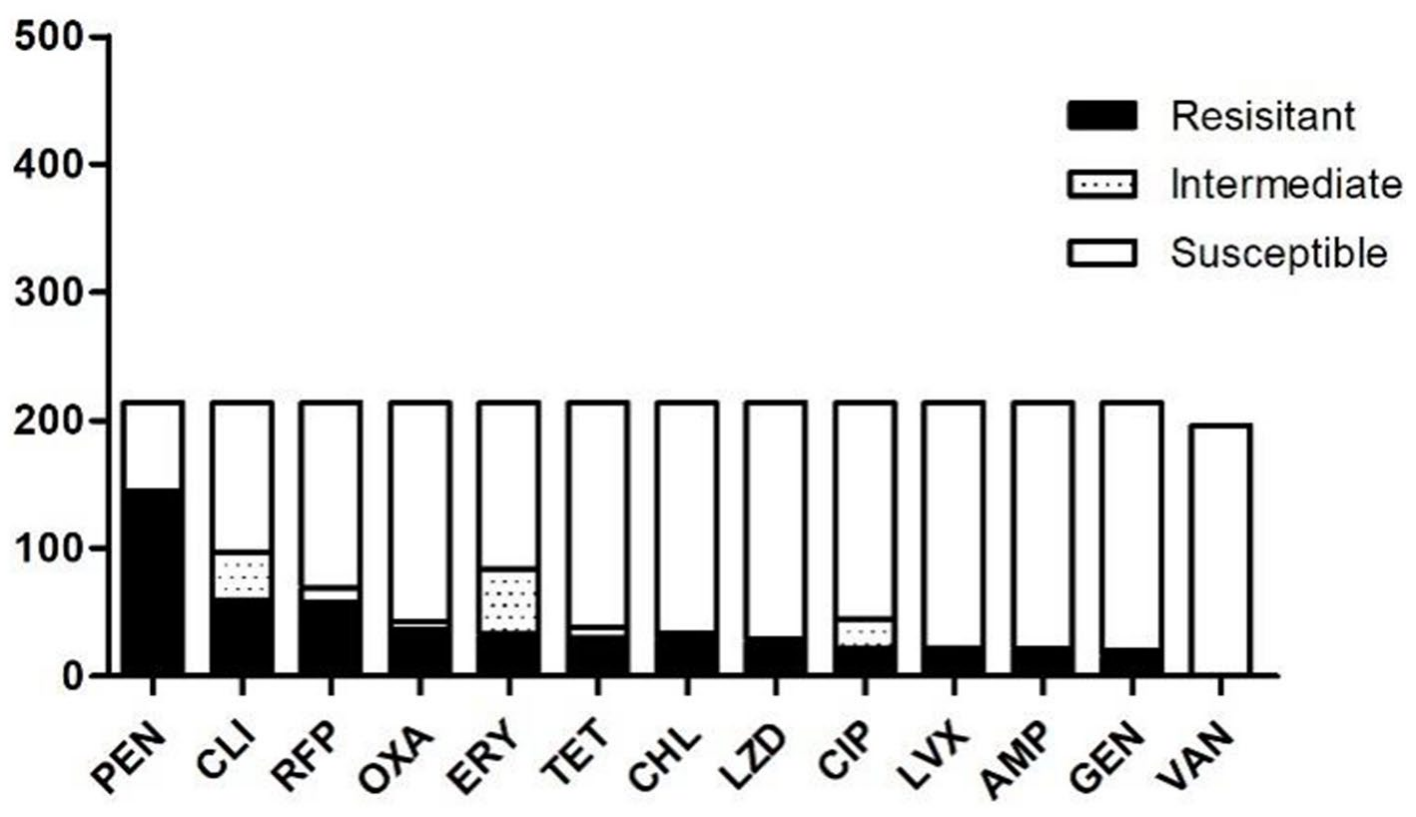
Profile of drug resistance of 214 strains of *S. aureus.* Black represents resistant, blank represents susceptible and hollow with dots means intermediate. PEN was the highest resistance, and all isolates were susceptible for VAN. Antimicrobial agents are abbreviated as follows: AMP = ampicillin; OXA = oxacillin; GEN = gentamicin; TET = tetracycline; CM = chloramphenicol; ERM = erythromycin; VAN = vancomycin; PEN = penicillin; RFP = rifampicin; CLI = clindamycin; ciprofloxacin, LVX = levofloxacin; LZD = linezolid.

A total of 214 strains of *S. aureus* were isolated. Figure 1 presents the results of resistance testing for 13 antibiotics. All strains exhibited resistance to at least one antibiotic. PEN exhibited the highest resistance, recorded at 67.76% (145/214). Subsequently, CLI recorded a percentage of 28.04% (60/214), followed by RFP at 27.10% (58/214). ERY exhibited a resistance rate of 15.42% (33/214), while AMP demonstrated a rate of 7.94% (17/214), and GEN showed a resistance rate of 7.01% (15/214).

The strains of *S. aureus* possessing the *mecA* gene were identified as MRSA. Among the 214 strains of *S. aureus*, 22 were identified as MRSA. The drug resistance profile was determined using 22 MRSA isolates and 22 randomly selected MSSA isolates (Supplementary Figure S1). The strains exhibiting the highest resistance to penicillin accounted for 36 instances, representing 23%, while those resistant to CLI totaled 26 instances, representing 17%. Four resistant strains exhibited drug resistance to LZD. Furthermore, 44 isolates yielded 31 distinct drug-resistant phenotypes (Table 2). Notably, 25 strains accounting for 58.14% exhibited multi-drug resistance (R ≥ 3). The SA8 strain demonstrated resistance to 11 antibiotics. The L8 strain exhibited an 8-fold increase in drug resistance. Four strains (SA9, SA7, SA10, and DP56) exhibited seven-fold resistance. The FF45 strain exhibited six-fold resistance. Eight strains—SA3, CP18, L1, EF45, SA17, SA5, DP29, and CF22—exhibited five-fold resistance. In addition, five strains exhibited 4-fold resistance, whereas nine strains demonstrated 3-fold resistance.

**Table 2:** Resistance profiles of 44 strains of *S. aureus* (*n* = 26).

Code	Phenotype	Number of drug resistance	Number	Percent/%
1	PEN-GEN-OXA-ERY-CIP-LVX-CLI-CHL-RFP-LID-VAN	11	1	2.32
2	PEN-ERY-CLI-CHL-RFP-LID-AMP-VAN	8	1	2.32
3	PEN-ERY-TET-CIP-LVX-CLI-RFP	7	1	2.32
4	PEN-ERY-TET-CIP-LVX-CLI-CHL	7	1	2.32
5	PEN-ERY-CIP-LVX-CLI-CHL-RFP	7	1	2.32
6	GEN-ERY-TET-CIP-RFP-LZD	6	1	2.32
7	GEN-CLI-FOX-PEN-TET	5	1	2.32
8	PEN-ERY-TMI-CLI	4	1	2.32
9	PEN-OXA-CLI-LID	4	1	2.32
10	CLI-FOX-PEN-TET	4	1	2.32
11	FFC-CIP- OXA-ERY	4	1	2.32
12	PEN-CAP-LVX	3	1	2.32
13	PEN-CLI-CHL	3	1	2.32
14	PEN-OXA-ERY	3	1	2.32
15	PEN-RFP-AMP	3	1	2.32
16	PEN-ERY-CLI	3	1	2.32
17	PEN-CLI-RFP	3	1	2.32
18	GEN-OXA-CLI	3	2	4.65
19	OXA-ERY-CLI	3	1	2.32
20	PEN-ERY	2	1	2.32
21	PEN-RFP	2	4	9.30
22	PEN-OX	2	1	2.32
23	PEN-CLI	2	2	4.65
24	GEN-CLI	2	1	2.32
25	PEN	1	10	23.26
26	GEN	1	4	9.30

**Figure 2:**
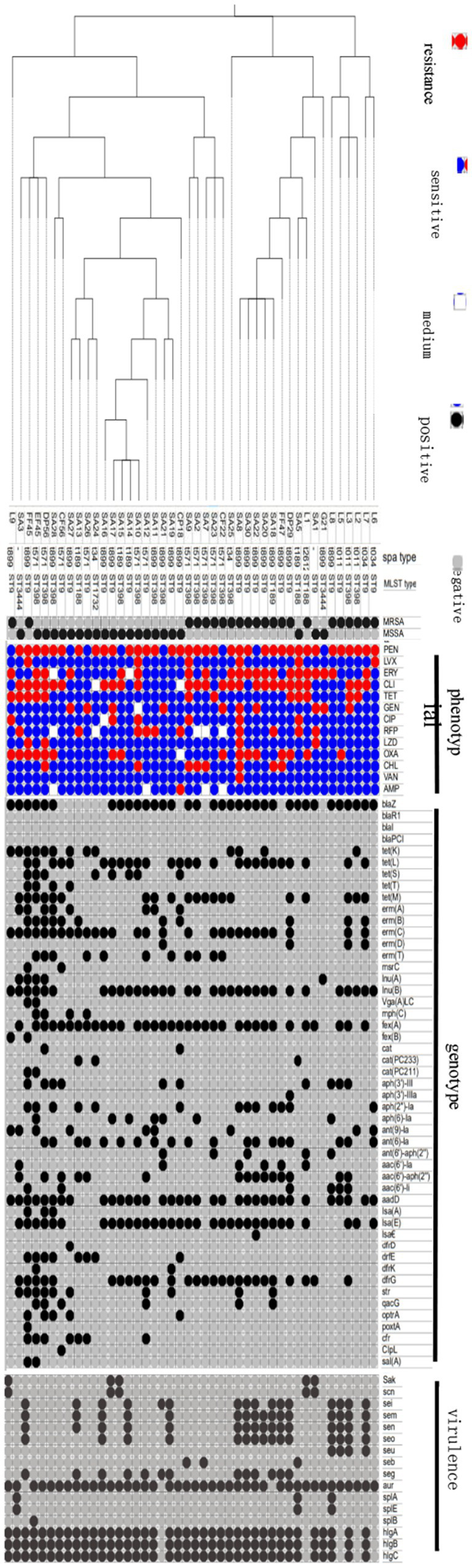
Antimicrobial resistance phenotype, genotype, virulence genes, molecular characteristics and phylogenetic analysis of 44 strains of *S. aureus* isolates. Forty-four isolates were classified into two types, MRSA and MSSA, in which 22 MRSA strains and 22 MSSA strains. The black dots represent positive and the brey represents negative. And antimicrobial resistance phenotype showed with three means medium. Antimicrobial agents are abbreviated as follows: AMP = ampicillin; OXA = oxacillin; GEN = gentamicin; TET = tetracycline; CM = chloramphenicol; ERM = erythromycin; VAN = vancomycin; PEN = penicillin; RFP = rifampicin; CLI = clindamycin; ciprofloxacin, LVX = levofloxacin; LZD = linezolid.

Figure 2 illustrates the drug resistance genes present in the isolates. All MRSA isolates possessed the *mecA* gene, while the *mecC* gene was absent, consistent with the resistance phenotype. The *blaZ, blaR1, blaI,* and *blaPCI* genes conferred resistance to penicillin. The genotype was consistent with the phenotype. The *tet(K), tet(L), tet(S), tet(T),* and *tet(M)* genes were responsible for TET resistance, with a direct correlation between the genotype and phenotype. The *erm(A), erm(B), erm(C), erm(D), erm(T), msr(C), lnu(A), lnu(G),* and *vga(A)* genes conferred resistance to MLSB antibiotics. However, the study identified resistance only to ERY and CLI. The *mph(C)* gene, responsible for encoding erythromycin phosphotransferase, was identified in the SA26, SA27, DP56, and EF45 strains. This gene conferred resistance to ERY in these strains. The genes *fex(A)* and *fex(B)* were associated with efflux pumps. The genes *cat(PC233)* and *cat(PC211)*, in conjunction with other genes, conferred resistance to chloramphenicol. The genotype was consistent with the phenotype. Aminoglycoside resistance was attributed to multiple genes. *Aph(3′)-III, aph(3′)-IIIa, aph(2″)-la, aph(6)-Ia, ant(9)-la, ant(6)-la, ant(6′)-aph(2″), aac(6′)-II,* and *aadD* represented various genetic elements associated with antibiotic resistance mechanisms. However, certain strains possessed resistance genes that did not completely correspond to their resistance phenotype. Three strains, SA28, DP56, and FF45, possessed the *lsa(E)* and *Isa(A)* resistance genes. The SA22 strain possessed *lsa(E)* and *lsa(€)*. A total of 28 strains possessed the *lsa(E)* gene. Linezolid resistance genes (*optrA*, *poxtA*, and *cfr*) were identified in nine strains: CP18, SA12, SA26, SA13, SA27, SA28, DP56, EF45, and FF45. Only four strains (SA1, SA8, DP56, and FF45) exhibited resistance to LZD. The *str* gene conferred resistance to streptomycin. The *dfrD, dfrE, dfrK,* and *dfrG* genes conferred resistance to trimethoprim. The *qacG* gene conferred resistance to quaternary amine disinfectants. Finally, the *ClpL* and *sal(A)* genes conferred resistance to ciprofloxacin; however, their role in drug resistance was not evaluated.

### 3.2 Molecular characteristics of the isolates

Figure 2 presents the results of SPA classification. A total of seven types were identified, with t899 being the most prevalent (19/43, 48.19%), followed by t571 (11/43, 25.59%), t189 (4/43, 9.30%), t34 (4/43, 9.30%), and t011 (3/43, 6.98%). T2612 was observed only once, accompanied by two unidentified types.

Figure 2 presents the results of MLST typing. A total of six sequence types (ST) were identified, with ST9 being the most prevalent (21/43, 48.84%), followed by ST398 (15/43, 34.88%), ST188 (3/ 43, 6.98%), and ST3444 (2/ 43, 4.65%). ST1732 was observed on a single occasion. The ST9 type (*n* = 22) was the most prevalent strain, and it is also identified as the dominant type in China. The rising prevalence of the ST398 type (*n* = 15) indicates a shift in the epidemic types present on domestic dairy farms. ST9-t899 was the predominant type, occurring 14 times. The organism exhibited the greatest quantity of virulence genes. The L8 strain possessed 11 virulence genes: *sei, sem, sen, seo, seu, aur, splA, splE, hlgA, hlgB,* and *hlgC*. Seven isolates exhibited nine virulence genes. Each of the remaining six isolates possessed four or more virulence genes. ST9-t899 (SA8) exhibited the greatest tolerance to 11 antibiotic types, while ST398-t571 (SA7) demonstrated tolerance to eight antibiotic types.

Figure 2 illustrates the prevalence of enterotoxin genes (*sak/scn, sei/sem/sen/eo/seu, seb/seg*), extracellular enzyme genes (*aur, spl A/B/E*), and hemolysin genes (*hlgA/B/C*) among the isolates. The L8 strain exhibited the highest number of virulence genes, comprising 11 distinct types. The L7, L3, DP29, FF47, SA22, SA30, SA8, SA19, SA14, and SA16 strains possessed nine virulence genes, while the L2 strain possessed a single virulence gene. The *aur* and *hlgA/B/C* genes were present in 41, 40, and 39 strains, respectively, with at least 90% of the strains exhibiting these four virulence genes. The *sei/sem/sen, seo*, and *seg* genes were identified in 16, 15, and 13 strains, respectively. The *splB* gene exhibited the lowest rate in the isolates, specifically in the L8 strain (Supplementary Figure S2).

### 3.3 Phylogenetic analysis

The isolates were classified into three primary clades: C1, C2, and C3, based on SNP and WGS analyses. The strains isolated from the identical cattle farm demonstrated specific clustering (Figure 2). The L6, L7, L2, L3, L5, and L8 strains isolated from household cattle farm 1 were classified within the C1 clade. Within the C2 clade, 67% (8/12) of the strains originated from intensive cattle farm 2, specifically SA1, SA5, SA18, SA20, SA22, SA30, SA8, and SA25. The remaining strains within the C2 clade consisted of L1 from household cattle farm 1, G21 from household cattle farm 2, and DP29 and FF47 from intensive cattle farm 1. Within the C3 clade, 57% (15/26) originated from intensive cattle farm 2. In addition, 23% (6/26) originated from intensive cattle farm 1, along with the L9 strain from the household cattle farm. In addition, 59% (26/44) of the strains were categorized within the C3 clade (Figure 2).

The C3 clade was divided into three subclades, with the DP56, EF45, and FF45 strains grouped within one subclade. This suggests cross-transmission between livestock and humans. The DP56 strain was identified as MSSA-ST398-t571, the EF45 strain was identified as MSSA-ST398-t34, and the FF45 strain was identified as MRSA-ST9-t899. These strains represent prevalent epidemic types observed in China. The resistance characteristics of the DP56, EF45, and FF45 strains are outlined as follows: DP56 exhibited resistance to ERY, CLI, TET, TEN, OXA, and CHL; EF45 demonstrated resistance to PEN, OXA, TET, RY, and CLI; and FF45 showed resistance to PEN, CLI, LVX, TET, OXA, and LZD. All strains exhibited multidrug resistance, demonstrating resistance to PEN, OXA, CLI, and TET. The FF45 strain exhibited resistance to LZD (Figure 2).

### 3.4 The biofilm formation capacity

**Figure 3:**
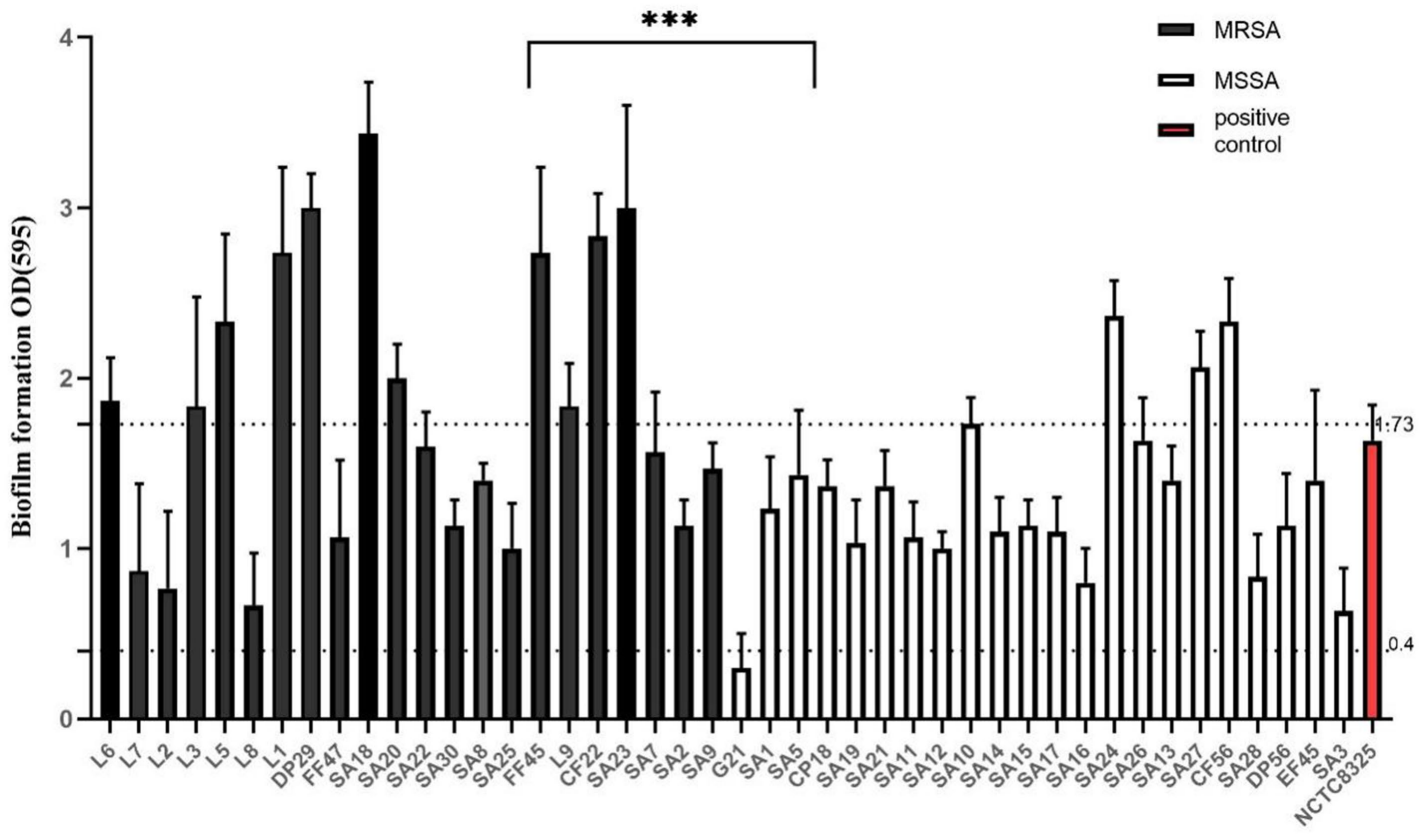
Biofilm formation capacity of NCTC8325 and 44 isolates. NCTC8325 was positive control. Forty-four isolates were divided into two groups, MRSA and MSSA. OD_595_ > 1.73 represented strong ability, 1.73 > OD_595_ > 0.4 represented moderate, OD_595_ < 0.4 represented weak. Error bars represented SD. ***p < 0.001.

Among the samples, 31.82% (14/44) exhibited strong characteristics, including 11 MRSA and three MSSA strains; 63.64% (28/44) displayed moderate characteristics, comprising 11 MRSA and 17 MSSA strains; and 2.27% (1/44) were classified as weak, represented by an MSSA strain (Figure 3). The majority of the strains exhibited moderate capabilities. MSSA isolates were significantly more prevalent than MRSA strains. The isolates exhibiting significant biofilm formation capacity were identified as MRSA. The MRSA and MSSA strains exhibited a statistically significant difference in biofilm formation (*t* < 0.001). These isolates demonstrated the potential to form biofilms, implying a considerable threat to livestock, the environment, and public health.

### 3.5 The result of the transferability experiment

Transformants containing the *poxtA* gene were obtained from the electrotransformation experiment. However, the *cfr* and *optrA* genes were absent in the transformants. This study found that the *optrA*-positive and *cfrA*-positive strains comprised 20.45% (9/44) of the total samples.

## 4 Discussion

The isolation rate of *S. aureus* in raw milk in the Jilin region was 42.80% (214 /500). The rate is lower than that reported in the Hangzhou region (60.70%) and the Shihezi region in Xinjiang (47.24%). However, this rate exceeds the figures reported in four regions of Henan Province (22.86%) and Gansu Province (30.11%) (Suzuki and Tagami, 2015; Liu et al., 2024; Kurz et al., 2024). This study identified that 10.28% (22/214) of MRSA isolates were present. The rate is lower than that observed in certain northern regions of China, where it was recorded at 27.70% (54/195) (Kadariya et al., 2014; Asati et al., 2024). This study found that penicillin exhibited the highest level of resistance, which aligns with the findings of Ahmad-Mansour et al. (2021) and Aires-de-Sousa (2017). The affordability of penicillin and its extensive application are acknowledged. However, the extensive use and misuse of antibiotics contribute to increasing drug resistance. The excessive and inappropriate use of antibiotics in livestock production has accelerated the emergence of AMR. Misuse of antibiotics in food-producing animals allows resistant bacteria to develop. These drug-resistant microbes can spread through the food chain, environment, and direct contact, posing severe threats to animal welfare, human health, and global ecosystem stability.

Linezolid has been used in China since 2007 for the treatment of MRSA infections. Current research indicates that the *cfr*, *optrA*, and *poxtA* genes are responsible for resistance to LZD. The majority of resistance genes are found on plasmids, which have a notably strong capacity for transfer. This ability is a critical mechanism for the dissemination of resistance among strains. The *optrA* gene exhibits the capacity for interspecies dissemination (Cuny et al., 2015; Matuszewska et al., 2020; Ferri et al., 2017). Genes resistant to LZD were identified in the DP56, EF45, and FF45 strains. The DP56 strain, which originated from a human, carried the *cfr* and *optrA* genes. The EF45 strain, derived from milk, carried both *cfr* and *optrA* genes. The FF45 strain, derived from milk, possessed the *cfr*, *optrA*, and *poxtA* genes. Although EF45 and FF45 were derived from the same cattle farm, they were located in different grids. Despite being managed by the same breeder, they were unable to come into direct contact. This demonstrated a connection between livestock and humans. The strain CC398 is now capable of transmission from humans to livestock. It has lost genes associated with human virulence and acquired resistance to methicillin and tetracycline (Ts et al., 2023). This study demonstrated that the FF45 isolate exhibited resistance to methicillin, tetracycline, and linezolid.

This study also observed that *poxtA* in MRSA (FF45) is capable of horizontal transfer, as shown in a transfer test. This could enhance the gene’s frequency within the population, potentially leading to higher resistance to linezolid. The genes *cfr* and *optrA*-positive were not detected in the transformants, which may be due to these genes typically being located on chromosomes rather than plasmids (Loffler and Tuchscherr, 2021).

Genes associated with linezolid resistance were identified in some strains within this study. The *optrA* gene was present in the CP18, SA27, and SA28 strains. The *cfr* gene was present in the SA12, SA26, and SA13 strains. The SA27 strain possessed both the *optrA* and *cfr* genes; however, none of these strains exhibited resistance to LZD. It is necessary to continue monitoring the potential role of cattle as a source of antibiotic genes.

Linezolid serves as the final therapeutic option for managing drug-resistant bacterial infections. However, it has not been applied in animal husbandry. The *optrA* gene confers resistance to oxazolidinone and amide alcohol drugs. The use of florfenicol, an antibiotic, may increase the selective pressure on this gene. This study demonstrated that MRSA strains exhibit resistance to linezolid. This resistance may be associated with the administration of oxazolidinone and amide alcohol medications. Antibiotic resistance genes (ARGs) are disseminated through mobile genetic elements (MGEs). Animals have the capacity to excrete these mobile genetic elements into the environment. Subsequently, they have the potential to infect other livestock and humans (Sarwar et al., 2023; Wang et al., 2012; Baoguang et al., 2019). Drug resistance to linezolid is frequently observed in pig farms; however, it has not yet been detected in milk samples. The genes associated with drug resistance and disinfectant resistance present in raw milk can potentially spread to humans and the environment. This transmission occurs through feeding practices, transportation equipment, and breeding facilities.

The *qacG* resistance gene was identified in six MRSA strains. It offers resistance to quaternary ammonium salt preservatives. Prior to 2015, the *qacG* gene had not been detected in 16 provinces of China (Qian et al., 2017). Research indicates that qacA/B contributes to the resistance of MRSA against disinfectants in China. This research identified the *qacG* gene, while the qacA and *qac*B genes were not detected. The resistance genes for quaternary ammonium disinfectants in Jilin exhibited variability. The six strains analyzed demonstrated resistance to multiple antibiotics, exhibiting resistance genes for *β*-lactam, tetracycline, MLSB, and chloramphenicol. The SA8, DP56, and EF45 strains exhibited the presence of CFR resistance genes. The plasmid containing the *qac* gene family in *S. aureus* can simultaneously harbor multiple resistance genes (Tingting et al., 2021; Shi et al., 2021). Disinfectants and preservatives are extensively utilized in the food industry. However, the impact of these factors on the development of disinfectant resistance genes or the dissemination of resistance genes remains uncertain. Therefore, enhancing the surveillance of disinfectant resistance genes is essential.

The DP56 strain possessed two genes associated with tetracycline resistance: Tn5397 and an additional tetracycline resistance protein. The organism carried two genes encoding the *blaZ* beta-lactamase, two genes for the ermT protein, and one gene for an unidentified product. The DP56 strain possessed the ß-lactamase gene; however, it exhibited sensitivity to ß-lactamase in preliminary drug resistance assessments. The results of the *mecA* gene test were negative. The discrepancy between the genotype and resistance phenotype of the DP56 strain may arise from the binding of the *β*-lactamase gene to various antibiotic sites. This may alter the function of the encoded protein (Liu et al., 2017). *Tet (M)* predominantly resides on the Tn5397 transposon within *Enterococcus*. It functions as a gene recombinase, operating both within and among populations (Chenqing et al., 2024). von Willebrand factor binding protein (VWB), known for its ability to agglutinate red blood cells in ruminants, pigs, and sheep, as well as to enhance pathogen pathogenicity, was identified in MRSA ST398 but was absent in the ST9 type (Rushun et al., 2022; Tang et al., 2021). In this study, the *vwb* gene was identified in the DP56 (ST398) and EF45 (ST398) strains, while it was absent in the FF45 strain (ST9). The *norB* gene in the DP56 strain encodes a multidrug efflux MFS transporter. *S. aureus* possesses the capability to express efflux pumps. This phenomenon may lead to resistance against disinfectants, macrolides, trimethoprim, and fluoroquinolones. Consequently, *S. aureus* is becoming an increasing concern in hospital- and community-acquired infections (Wang et al., 2024; Tang et al., 2023). The iron regulatory gene can be horizontally transferred between various strains via the original bacteriophage, thereby augmenting its pathogenic potential (Price et al., 2012). The DP56 strain contains this gene, whereas the EF45 and FF45 strains do not possess iron regulation-related genes.

The biofilm produced by *S. aureus* serves as a significant virulence factor that allows the bacteria to endure variations in temperature, pH, and other unfavorable environmental conditions (Zhu et al., 2020). This study found that all 44 strains were capable of biofilm formation, albeit at varying densities. Our findings indicate that MRSA isolates exhibit a significantly greater capacity for biofilm formation compared to MSSA strains. The drug resistance of MRSA strains is inherently associated with their capacity to form biofilms. The factors influencing bacterial drug resistance are diverse, including nutritional limitations, osmotic barriers, and quorum sensing. Biofilm formation alters bacteria in ways that make them largely resistant to the majority of antibiotics. Bacteria with biofilms exhibit significant resistance to pharmacological agents (Gao et al., 2020; Ondon et al., 2021).

The increase in antimicrobial resistance (AMR) poses a significant threat to global health; therefore, monitoring AMR from a One Health perspective is crucial. Novel drug-resistant genes, disinfectant-resistant genes, and molecular traits continue to emerge in the Jilin region. Contamination has been observed in *optrA*-positive, *cfrA*-positive, and *poxtA*-positive isolates. Notably, the plasmid *poxtA* found in the EF45 strain demonstrated the capacity for horizontal transfer. Genetic analysis indicated transmission from humans to livestock. Tracking drug resistance and molecular traits is essential for managing *S. aureus*, particularly MRSA, and for ensuring food safety.

## Data Availability

The original contributions presented in the study are included in the article/Supplementary material, further inquiries can be directed to the corresponding author.

## Footnotes

^1^ www.genomicepidemiology.org/services
