# Supplementary material for: Genomic epidemiology and characterization of Staphylococcus aureus isolates from raw milk in Jilin, China: evidence of contamination with optrA-positive, cfrA-positive, and poxtA-positive strains and human–livestock transmission
Source: Front Microbiol. 2025 May 29;16:1547283. doi: 10.3389/fmicb.2025.1547283 (PMC12158939; doi:10.3389/fmicb.2025.1547283)
Supplement: Supplementary file 1 [file Data_Sheet_1.docx]

Supplementary table 1. All primers.

| Gene | Sequence (5’--3’) | Product size(bp) |
| --- | --- | --- |
| *16SrRNA* | AACTCTGTTATTAGGGAAGAACA  CCACCTTCCTCCGGTTTGTCACC | 756 |
| *nuc* | GCGATTGATGGTGATACGGTT  AGCCAAGCCTTGACGAACTAAAGC | 279 |
| *mecA* | GTAGAAATGACTGAACGTCCGATAA  CCAATTCCACATTGTTTCGGTCTAA | 533 |
| *mecC* | A A GTTAATCAAAAATGGGTTCAGC  GGTTGTAATGCTGTACCAGATCC | 568 |
| *poxtA* | GGTCTGACTGGCTTGTTTTGCT | 769 |
|  | ATAAGGTCGGTATTGTCGGCGT |  |
| *cfr* | TGAAGTATAAAGCAGGTTGGGAGTCA | 746 |
|  | ACCATATAATTGACCACAAGCAGC |  |
| *optrA* | AGGTGGTCAGCGAACTAA | 1395 |
|  | ATCAACTGTTCCCATTCA |  |

Supplementary table 2. PCR amplification system.

| Ingredient | 含量 |
| --- | --- |
| dd H_2_O | 9.50 μL |
| Bacterial lysate | 1.00 μL |
| *mecA* F(10 μmol) | 1.00 μL |
| *mecA* R(10 μmol) | 1.00 μL |
| PCR mix | 12.50 μL |
| Total | 25.00 μL |


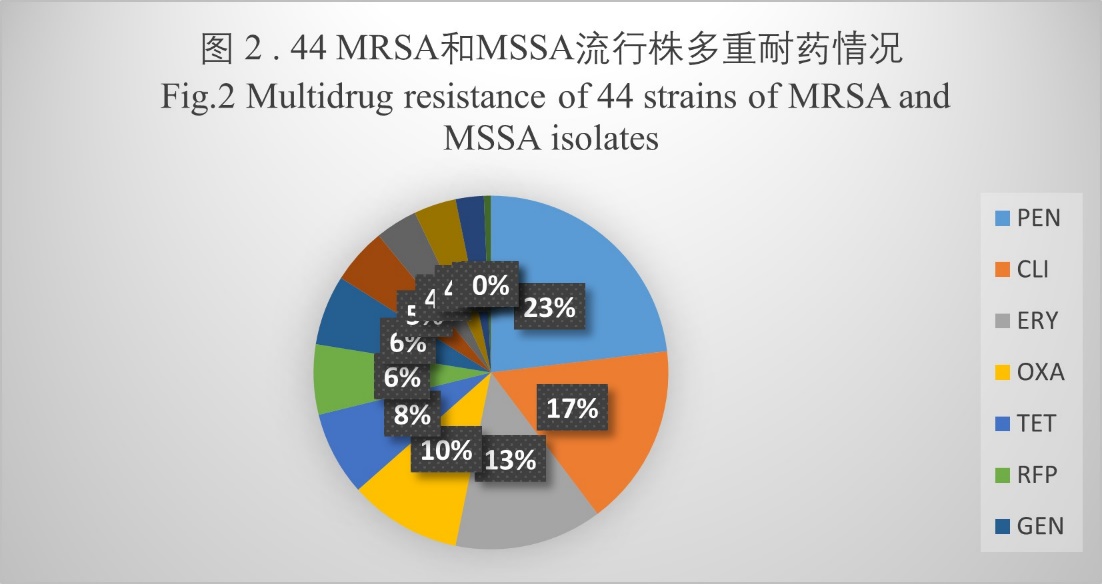


Supplementary Fig. 1. Drug resistance analysis of 24 MRSA and 20 MSSA
epidemic strains


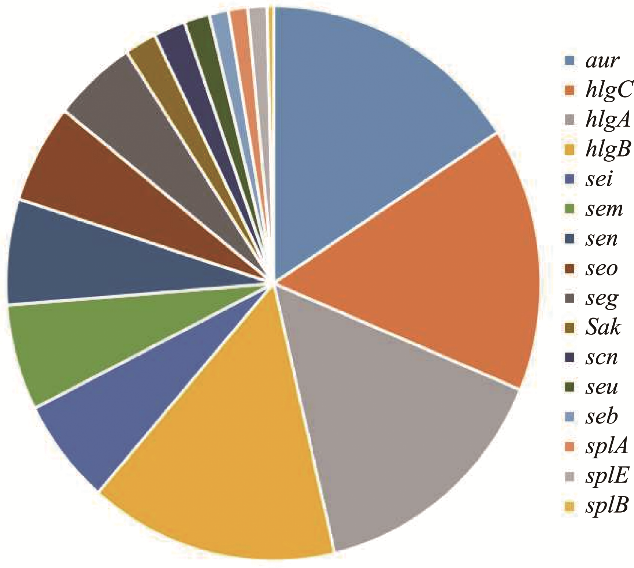


Supplementary Fig.2 Distribution of virulence genes in 44 MRSA and MSSA strains
